# Synthesis and Characterization of Pyridine Acetohydrazide Derivative for Antimicrobial Cotton Fabric

**DOI:** 10.3390/ma16134885

**Published:** 2023-07-07

**Authors:** Saeed El-Sayed Saeed, Meaad Aldubayyan, Ahmed N. Al-Hakimi, Marwa M. Abd El-Hady

**Affiliations:** 1 Department of Chemistry, College of Science, Qassim University, Buraidah 51452, Saudi Arabia; 2 Department of Chemistry, College of Science, Ibb University, Ibb 70270, Yemen; 3 National Research Centre, Institute of Textile Research and Technology, Dokki, Giza P.O. Box 12622, Egypt

**Keywords:** pyridine acetohydrazide, cotton coating, metal complex, ligand, antimicrobial activity

## Abstract

An increase in textile resistance to antimicrobial agents has posed a pressing need for the development of new antimicrobials. Therefore, the antimicrobial characteristics of thiophene and pyridine acetohydrazide derivatives have been developed as novel textile-modified complexes exhibiting antibacterial agents. Synthesis and characterization of pyridyl-thienyl acetohydrazide derivative (AHZ) using NMR (^13^C and ^1^H) and FTIR. Modification of cotton fabric (CF) with acetohydrazide (AHZ) and metal chlorides of divalent Cr, Mn, Co, Ni, Cu, and Zn and trivalent Fe, and Cr. SEM-EDX and Fourier-transform infrared were utilized to characterize cellulose-based cotton fabric (CF) attached to AHZ and their metal (M) complexes. Antimicrobial activity was examined against two types of bacteria, namely *S. aureus* and *E. coli*, and two types of fungi, namely *C. albicans* and *A. flavus*. All modified samples exhibited higher efficiency towards bacterial strains than fungal strains. In addition, cellulose modified with Ni (II) confers the most antibacterial protection efficiency.

## 1. Introduction

Due to the increasing number of microorganisms that are resistant to antibiotics, there is a significant need to develop novel antibacterial agents other than commercially available compounds to combat this [1]. Cellulose, which is the main component of cotton fabric, is the most common type of natural polysaccharide derived from algae, trees, plants, and bacteria. Cotton is a biodegradable, natural, hydrophilic cellulose-based fiber with OH functional groups that has numerous benefits in textile and biomedical engineering [2,3]. Cotton fabrics encourage the growth of germs like bacteria and fungi [4]. The most promising area for new textile materials is medical fabrics with antibacterial properties. Significant efforts are being made to enhance materials and techniques that could provide secure and efficient defense from various bacteria [5]. Therefore, it is essential to understand cotton fabrics’ (CF) antibacterial properties. Numerous antibacterial materials have been imparting antibacterial properties and improving them, including nanomaterials [6], reduced graphene oxide/silver nano complex [7], chitosan [8], and curcumin/titanium dioxide nanocomposite [5].

There is a lot of curiosity about the biological activity of hydrazides (R-CO-NH-NH_2_), such as their antibacterial, antifungal, and antitumoral effects [9]. Many ligands of amino acids hydrazide derivatives have been reported to act as models for biologically significant species such as metalloenzymes, making them vital in the advancement of bioinorganic chemistry. These complexes are well-known for their outstanding functions in biological, analytical, therapeutic, and industrial applications in addition to their vital roles in catalysis, drug dealing, and chemical synthesis [10]. Recently the ability of complexes of acetohydrazide derivatives to interact with DNA more than the free ligands has been demonstrated [11].

Because of their different biological activities, pyridine ring systems are very important classes of compounds. Biological targets include viral infections, different malignant cells, and microbial diseases when substituents on the pyridine nucleus are modified. To target specific biological problems, these compounds interact with enzymes, proteins, and DNA [12,13].

Cysteine, glutathione (GSH), taurine, methionine, *N*-acetylcysteine (NAC), and other sulfur compounds are all amino acids that include sulfur, making it a vital component in regular physiological function [14]. Othman et al. (2020) created many pyridine compounds, including the (imidazo [4,5-b]thieno [3,2-e]pyridine-6-carboxylic acid) and its ketone derivatives, which showed promising activity against all strains, particularly *K. pneumoniae*, *A. flavus*, and *A. ochraceus* [1]. To continue our research on cellulose treatment and applications [15,16]. In our previous textile applications Schiff base metal (M) complexes that modified cotton fabric (CF) were studied and showed promising antimicrobial activities [17,18]. Also, cadmium pyrimidinethione hydrazide-modified CF was studied and produced more antimicrobial than the other metal (M) [19]. In this work, the synthesis of 2-((3-cyno-4-(4-methoxyphenyl)-6-(thiophen-2-yl)pyridine 2yl) oxy)acetohydrazide (AHZ) ligand is characterized using different techniques. CF was treated with the synthesized AHZ, which was subsequently reacted with Zn (II), Ni (II), Cr (III), Co (II), Cu (II), Mn (III), or Fe (III) chlorides to form CF modified metal complexes. The antimicrobial effectiveness of the treated fabrics was evaluated.

## 2. Materials and Methods

### 2.1. Materials

Mill-bleached pure 100% cotton fabric was procured from Mehalla El-Kobra, Egypt, by Misr Company for spinning and weaving.

### 2.2. Chemicals

The starting chemicals were metal salts, ammonium acetate, hydrazine hydrate, acetyl thiophene, 4-methoxy benzaldehyde, ethyl cyanoacetate, ethyl chloroacetate, and potassium carbonate that were purchased from Sigma Aldrich and utilized without additional purification. Fisher Scientific (Loughborough, UK) supplied the solvents, which were ethanol (99%) and *N,N*-Dimethylformamide (99%).

### 2.3. Syntheses of Acetohydrazide Ligand (AHZ)

**Scheme 1:**
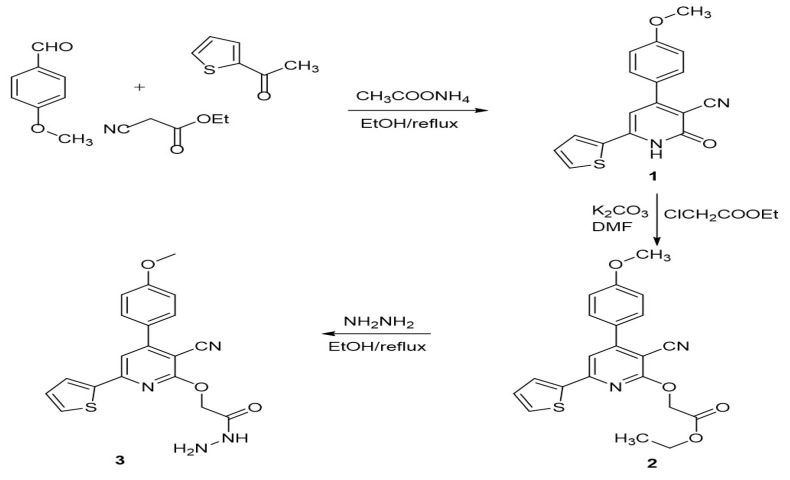
Preparation of pyridyl-thienyl acetohydrazide derivative.

AHZ ligand was prepared as in our previous work [20] using the following steps: (Scheme 1)

1st step: Preparation of 4-(4-Methoxyphenyl)-2-oxo-6-(thiophene-2-yl)-1,2-dihydropyridine-3-carbonitrile (1)

In Et OH (40 mL), 5 mmol of (acetyl thiophene and 4-Methoxybenzaldehyde), 15 mmol of ammonium acetate were added to 5 mmol of ethyl cyanoacetate, and the mixture was heated under reflux while stirring for five hours. The reaction mixture was cooled to 25 °C. The precipitated was then washed with ethanol, dried, and recrystallized from EtOH-DMF mixture to yield **compound 1** as a yellowish powder [21].

2nd step: Preparation of Ethyl 2-((3-cyano-4-(4-methoxyphenyl)-6-(thiophen-2-yl)pyridin-2-yl)oxy)acetate (2)

A solution of Ethyl chloroacetate and potassium carbonate (5 mmol: 5 mmol) was added to the substituted cyanopyridine derivative (1) that had been thoroughly stirred for 30 min. At 25 °C, the reaction mixture was stirred once more for six hours. The created solution was poured over ice-cold water. The ethyl **ester derivative 2** was produced as a yellowish powder by filtering, washing with water, drying, and crystallizing the precipitate that resulted from an EtOH-H_2_O (2:1) mixture.

3rd step: preparation of AHZ ligand (3)

Hydrazine hydrate (99%) was added to a solution of the ester 2 (5 mmol) in absolute EtOH (25 mL), and the solution was refluxed for 4 h. The solvent was removed using a rotary evaporator. The resulting precipitate was filtered, dried, and crystallized to produce the yellow crystals of AHZ **compound 3**.

### 2.4. Coating of Cotton Fabric (CF) with AHZ and M-AHZ

For 3–4 min at (50 °C, 40 kHz), 0.1 g of the prepared ligand was sonicated in thirty ml of DMF. In total, 1 g of cotton textile was soaked in the previous solution and sonicated for 30 min. Samples were then dried and produced cotton fabric-based cellulose modified with acetohydrazide (AHZ-CF). After that, 0.1 g of metal salt (Zn, Ni, Cu, Co, Cr, Mn, or Fe) was added to the mixture. Once again, the AHZ-CF sample was submerged in solution while being stirred constantly under sonication for 30 min. After removal, washing in distilled water, and drying, the resulting cotton fabric (CF) treated with AHZ metal complexes was obtained (M-AHZ-CF); M is Co, Cr, Mn, Cu, Fe, Ni, or Zn) [17].

### 2.5. Instruments

The ^1^H and ^13^C NMR spectroscopies were carried out using a Bruker spectrometer at 850 MHz (Billerica, MA, USA). The solid-state Fourir-transform infrared (FTIR) spectra were carried out using an Agilent spectrometer (Cary 600) in the 4000–400 cm^−1^ wavenumber range (Santa Clara, CA, USA). An SEM analysis was implemented by means of a VEGA3 (Tescan, Brno, Czechia). An energy-dispersive X-ray (EDX) was achieved by using JEOL JSM-7100F (EDX, Oxford X-act, Tokyo, Japan).

### 2.6. Tensile Strength

The test method (ASTM D-1682-94, (1994) [22] was utilized to determine the tensile strength of the fabric specimens. Two specimens were examined in the warp direction to measure the breaking load (Lb) of each modified fabric, and the average value was reported.

### 2.7. The Add-On (%) Loading

The equation was used to calculate the add-on

$$Add-on\left(\%\right)=\frac{W_{2}-W_{1}}{W_{1}}\times 100$$

where (*W*_1_) and (*W*_2_) are the pre and post treatment weights of specimens of the CF, respectively.

### 2.8. Antimicrobial Efficiency

The coated fabrics with the AHZ and M-AHZ were tested for antimicrobial activity against various strains of Gram-negative bacteria *Escherichia coli* (*E. coli*) and Gram-positive bacteria *Staphylococcus aureus* (*S. aureus*), as well as fungal strains *Candida albicans* (*C. albicans*) and *Aspergillus flavus* (*A. flavus*) using the disk diffusion method [5].

## 3. Results and Discussion

### 3.1. Characterizations of AHZ Ligand

#### 3.1.1. NMR

**Figure 1:**
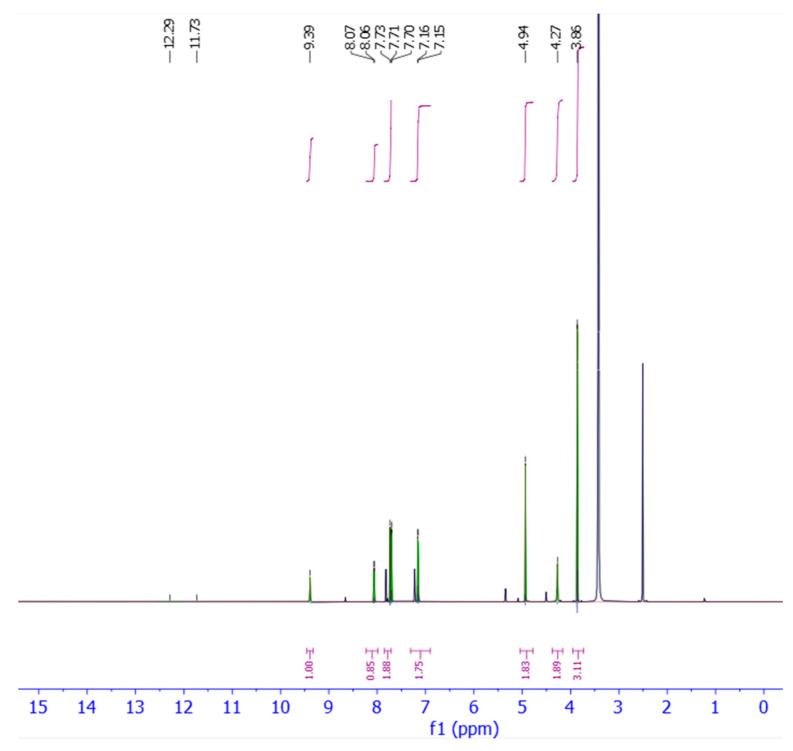
The ^1^HNMR of AHZ ligand.

**Figure 2:**
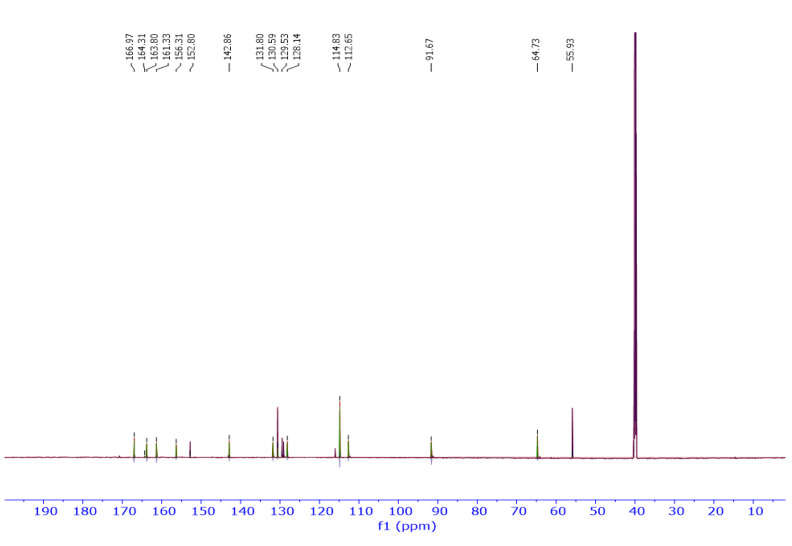
The ^13^CNMR of AHZ.

Characterization of the AHZ ligand Yield: 82%; m.p. 196 °C; IR (KBr, cm^−1^) υ: 3324–3138 (NH_2_, NH), 1676 (C=O), 3050 (C–H ar), 2960 (C–H aliph), 2218 (CN), 1610 (C=N ar), 1021 (N–N), 819 (C–S). ^1^H NMR (DMSO-d6) (Figure 1) δ: 3.87 (s, 3H, OCH_3_), 4.27 (s, 2H, NH_2_), 4.93 (s, 2H, CH_2_), 7.16 (d, 2H, J = 8.4 Hz, Ar–H), 7.23 (m, 1H, Ar–H), 7.73 (d, 2H, J = 7.8 Hz, Ar–H), 7.75 (d, 1H, J = 8.2 Hz, Ar–H), 7.83 (s, 1H, pyridine-H5), 8.05 (d, 1H, J = 7.6 Hz, Ar–H), 9.39 (br, 1H, NH). ^13^C NMR (Figure 2) (DMSO-d6) δ: 55.9 (CH_3_), 64.7 (CH_2_), 91.6 (CN), 112.2, 112.7, 114.8, 116.0, 128.9, 129.1, 129.5, 131.8, 142.9, 143.1, 152.8, 161.3, 163.8 (Ar–C, pyridyl-C, thienyl-C), 166.4 (C=O). Elemental analysis (%): calcd. for C19H16N4O3S: H, 4.24; C, 59.99; N, 14.74; S, 8.43. Found: H, 3.76; C, 54.45; N, 7.23; S, 20.91. λmax (nm): 300 and 241.

#### 3.1.2. FTIR Spectra of AHZ

**Figure 3:**
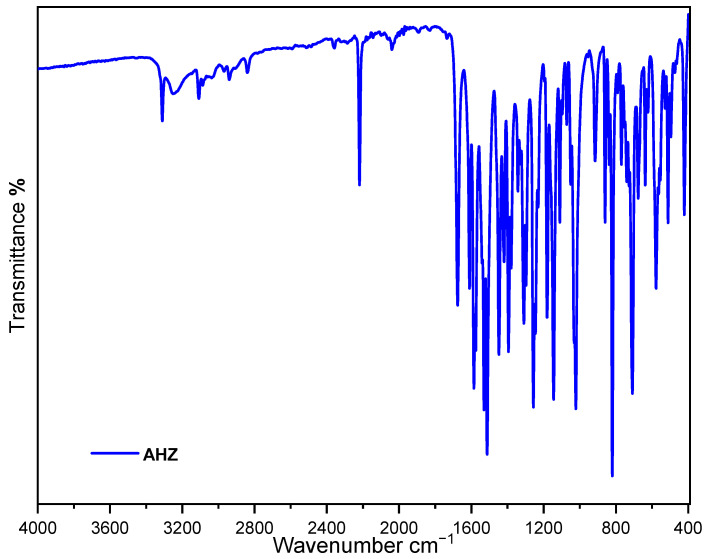
The FTIR of AHZ.

The ligand’s significant FT-IR spectral bands are shown in Figure 3. The spectrum of the AHZ appeared medium band in the range of (3183–3324 cm^−1^), which is conformable to NH_2_ and NH groups, and a strong band at 2218 cm^−1^ concerning the C≡N group [23], whilst the strong band at 1676 cm^−1^ concerning the C=O group of acetohydrazide [9], and strong bands at 1610, 1021 cm^−1^ concerning C=N and N–N of the pyridine ring and acetohydrazide [24,25], respectively. At 819 cm^−1^, a strong band induced by (C–S) thiophene stretching appeared [26].

### 3.2. Cotton Fabric Analysis

#### 3.2.1. The Proposed Mechanism between CF and AHZ Derivative

**Scheme 2:**
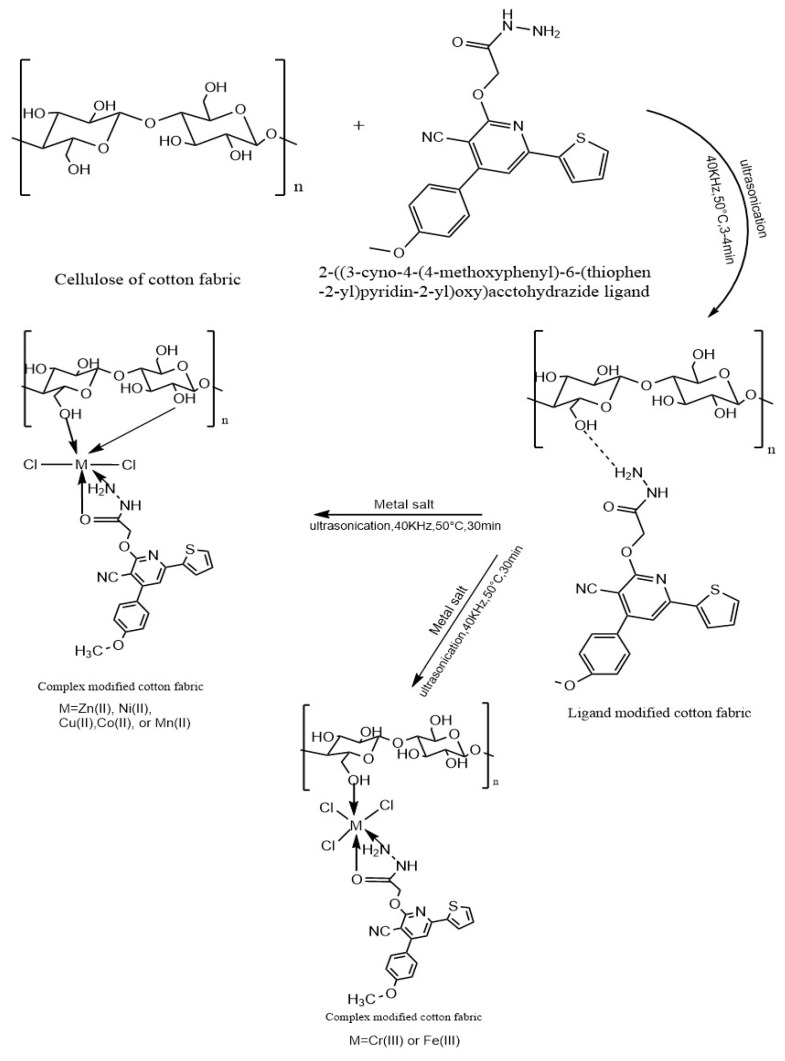
The proposed interaction mechanism between the cellulose-based CF and metal complexes.

Scheme 2 shows the suggested interaction between the cellulose CF and the acetohydrazide complex. The acetohydrazide ligand and cotton fabric cellulose molecules interact primarily through H-bonding and a weak Vander-Wall interaction between the amino group of the AHZ and the OH groups of the cellulose molecules. Upon complexation, the participation of the C=O, NH_2_ of the AHZ molecule, and OH group of cellulose chains in binding to the metal ions. Coordination bonds are created between the OH groups of cellulose chains and metal ions. As a consequence, a complex is created between AHZ and the cellulose structure, using the metal ions attached in the cellulose chain via coordination bond.

#### 3.2.2. FT-IR Spectra of the Modified Cotton

**Figure 4:**
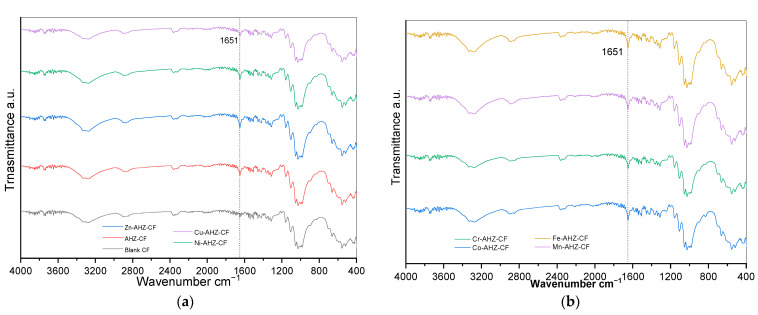
(**a**) and (**b**) FTIR of blank CF, ligand coated CF, and metal complexes coated CF.

The adsorption of AHZ and M-AHZ on the surface of CF is studied using the FT-IR spectral method. Figure 4 and Table 1 show the spectra of blank cotton fabric, as well as AHZ and complex modified CFs. A peak appeared in the (3200–3500 cm^−1^) range for unmodified CF, which can be assigned to O–H stretching. The cellulose vibrations of C–H stretching and C–H bending were attributed to the weaker peaks at 2904 cm^−1^ and 1371 cm^−1^ [27]. While an appearance of O–H, C–O–C, and C–O vibrations caused the cellulose bands to appear in the 1500–800 cm^−1^ range [7]. Moreover, the spectra of modified CFs showed characteristic peaks related to cellulose structure, in addition to the peaks of the AHZ. Figure 4 shows that AHZ-CF has a new peak at 1652 cm^−1^, which corresponds to the stretching C=O group of the ligand. Furthermore, AHZ-CF has broadband around 3468 cm^−1^ and 3191 cm^−1^ [28], which could be due to vibrational (NH) stretching vibration for a ligand latent behind the hydroxyl band CF. In this instance, hydrogen bonding dominates the interaction between the acetohydrazide ligand and the cellulose molecule, with a minor Van-der-Wall interaction between the NH_2_ group of the AHZ and the hydroxyl groups of the cellulose structure. C=O, NH_2_, and OH groups are shifted to lower wavenumbers due to complexation with metal ions [29,30]. The participation of the NH_2,_ C=O of the AHZ, and the OH group of cellulose in binding to the metal ion is supported by these shifts. Furthermore, all metal complexes have peaks M–Cl, M–O, and M–N in the range of 592 to 434 cm^−1^ which overlap with peaks of CF [31,32].

#### 3.2.3. SEM/EDX Analysis

**Figure 5:**
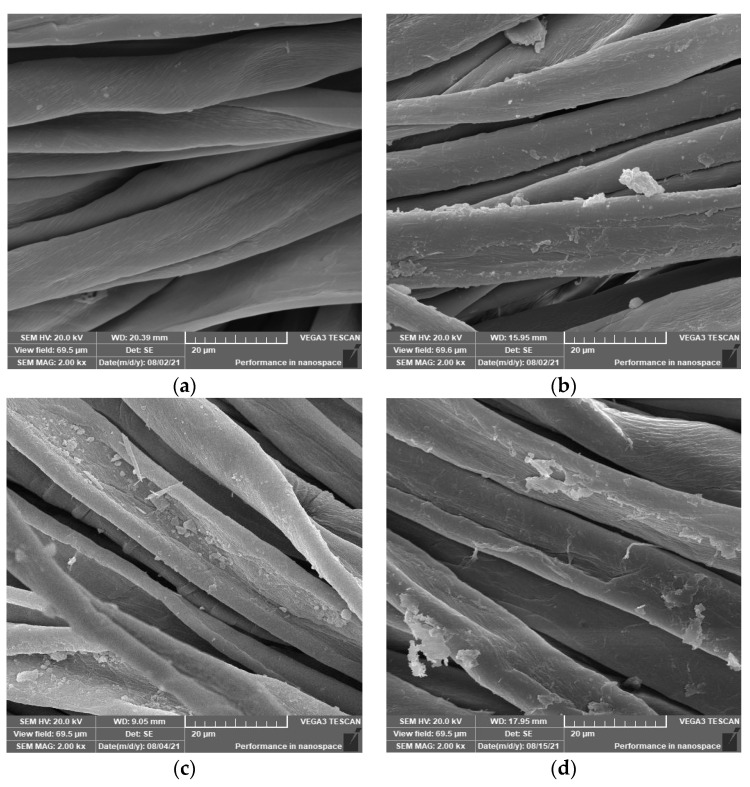

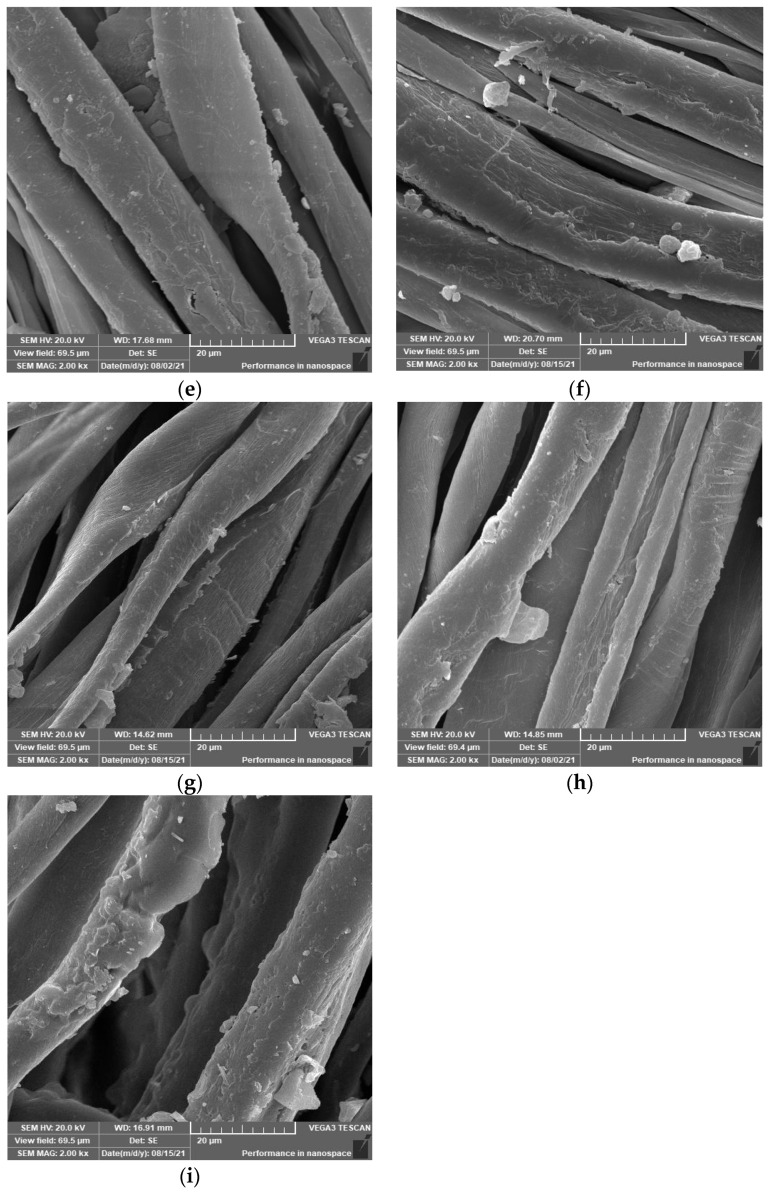
SEM images of untreated CF (**a**) and the treated fabric; AHZ-CF (**b**), Ni–AHZ-CF complex (**c**), Cu-AHZ-CF complex (**d**), Zn–AHZ-CF-complex (**e**), Co–AHZ-CF complex (**f**), Cr–AHZ-CF complex (**g**), Mn–AHZ-CF complex (**h**), and Fe–AHZ-CF complex (**i**).

**Figure 6:**
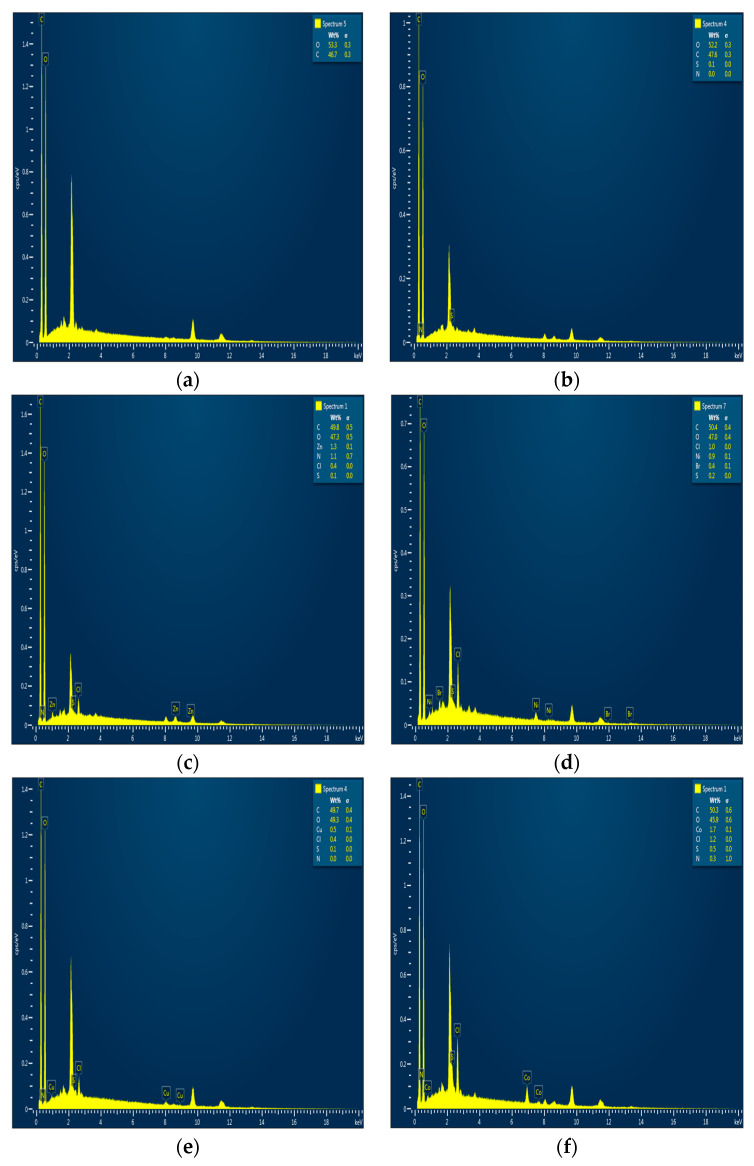

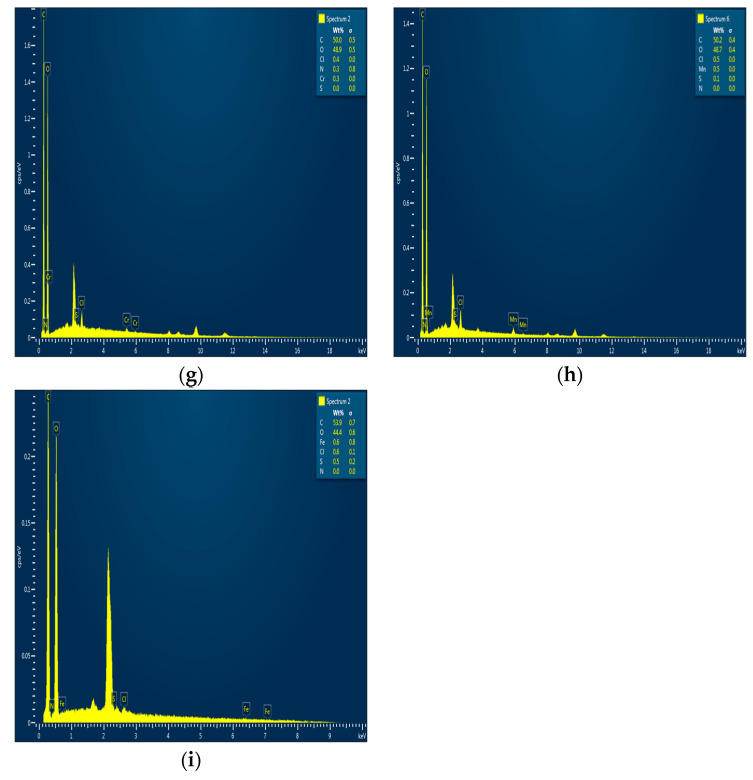
The EDX analysis of modified cotton fabric with ligand and metal complex, where blank (**a**), AHZ-CF (**b**), Zn−AHZ-CF complex (**c**), Ni−AHZ-CF complex (**d**), Cu−AHZ-CF complex (**e**), Co−AHZ-CF complex (**f**), Cr−AHZ-CF complex (**g**), Mn−AHZ-CF complex (**h**), and Fe−AHZ-CF complex (**i**).

The surface morphology of cotton fabrics (CF) with acetohydrazide ligand and its metal complexes were evaluated using scanning electron microscopy (SEM). Figure 5a displays the SEM of the blank cotton fabric, which reveals the appearance of a smooth surface [33,34], whereas Figure 5b–i shows agglomerated particles on the coated cellulosic fiber surface (CF) with acetohydrazide (AHZ) and the CF modified with acetohydrazide metal complexes (M–AHZ-CF; M is Cu, Ni, Zn, Co, Cr, Mn, or Fe) as a result of the modification of CF. Figure 6 also shows the results of an EDX analysis of coated cellulose cotton fabric (CF) with acetohydrazide ligand (AHZ) and AHZ metal complexes. The untreated fabric (Figure 6a) demonstrates that only carbon and oxygen are present in the fabric’s composition. The deposition of the acetohydrazide on the cellulosic fiber is indicated by the appearance of new elements in Figure 6b. Figure 6b–i show Zn, Ni, Cu, Co, Cr, Mn, and Fe metals with nitrogen and sulfur, which is evidence that the CF was successfully modified with AHZ metal complexes [34].

#### 3.2.4. Antimicrobial Studies

**Figure 7:**
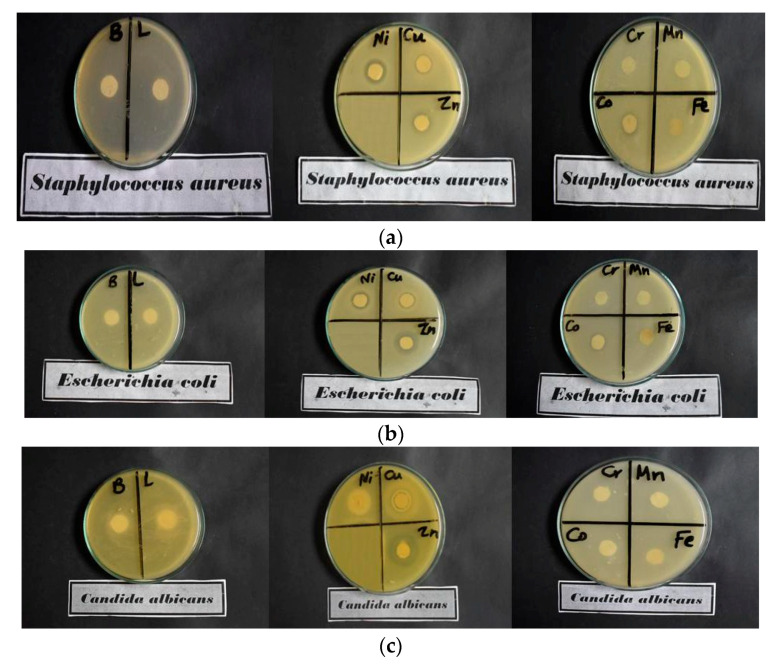

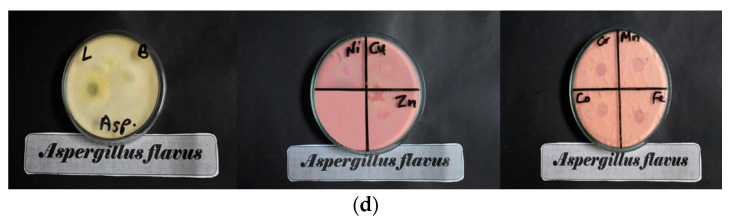
Inhibition zone of blank and coated cotton fabrics against *S. aureus* (**a**), *E. coli* (**b**), *C. albicans* (**c**), and *A. flavus* (**d**).

**Figure 8:**
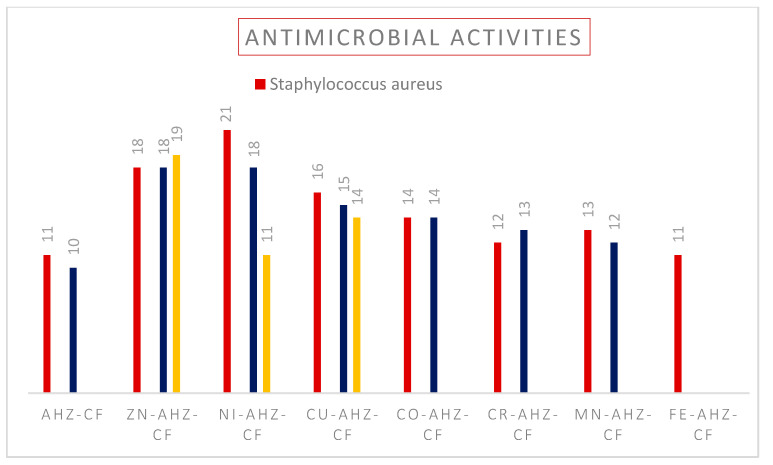
The antibacterial and antifungal activities of all coated cotton fabrics.

Using the disk diffusion method, the microbial activity of unmodified and modified CFs were tested against representative pathogens, including Gram+ (*S. aureus*) and Gram- (*E. coli*). Also, all samples were tested against a variety of fungal strains, namely (*A. flavus*) and (*C. albicans*). As shown in Table 2 and Figure 7, all treated samples of cotton fabric had an antibacterial action with a zone of inhibition ranging from 10 to 21 mm. It is clear from Table 2 that all modified CFs with AHZ and its M–AHZ complexes had almost a greater response to Gram-positive than Gram-negative, and variance in bacterial cell wall organization structure may account for this. It was noticed that the ligand modified cotton fabric was found to have moderate activity against Gram-positive (*S. aureus*) bacteria, whereas low activity against Gram-negative (*E. coli*) bacteria. When compared to the AHZ-CF, cotton fabric modified with metal complexes was found to have remarkable antimicrobial activity. The Ni–AHZ-CF complex demonstrated the highest antibacterial activity against both Gram- and Gram+ bacteria, followed by the Zn–AHZ-CF, as well as the Fe–AHZ-CF displayed the lowest antibacterial activity when compared to the other M−AHZ-CF. The order of antibacterial effects is as follows: Ni–AHZ-CF > Zn–AHZ-CF > Cu–AHZ-CF > Co–AHZ-CF > Cr–AHZ-CF, Mn–AHZ-CF > Fe–AHZ-CF, as presented in Figure 8. The improved performance of M-AHZ complexes can be demonstrated by the chelation theory [35,36,37]. This indicated that upon complexation, the polarity of the metal ion is diminished as a result of its positive charge being shared partially with donor groups. In addition, it boosts the complex’s lipophilicity and increases the delocalization of π-electrons across the entire chelate ring. This increased lipophilicity improves the complexes’ ability to penetrate the bacterial cell membrane’s lipid layer and hinders the M-binding sites on the enzymes of microorganisms. On the other hand, Table 2 also shows the antifungal activity of all treated samples. Ligand-modified cotton fabric has no activity against both fungal strains, (*A. flavus*) and (*C. albicans*). Metal complexes modified cotton fabric were found to have a higher activity against *C. albicans* than *A. flavus*. All complexes had no activity against *A. flavus*. Zn–AHZ-CF was found to possess higher antifungal activity against *C. albicans* compared with all the studied samples of cotton fabric-modified complexes (Figure 7).

#### 3.2.5. The Add-On (%) and Tensile Strength

The add-on values represent the amount of chemicals used during the modification process that were deposited on the CF. Table 3 shows add-on percentage results. The outcomes presented that the add–on values for modified CF with AHZ were 2.1% while the M-AHZ modified fabrics show much larger add–on values ranging from 4.5% to 9.4%. In contrast, the treated samples’ tensile strength values have significantly decreased. This could be explained by the various modifications made to cotton fabric.

## 4. Conclusions

Herein, we report the synthesis of 2-((3-cyano-4-(4-methoxyphenyl)-6-(thiophen-2-yl) pyridin-2-yl) oxy) acetohydrazide (AHZ). Spectroscopic techniques including ^1^HNMR spectra, ^13^CNMR spectra, and FTIR spectra successfully elucidated the AHZ structure. The CF were modified with the AHZ and its complexes by exposing them to ultrasonic waves for several minutes. SEM images represent distributions of the AHZ and M-AHZ on the surface of fabric resulting in a rough surface. The EDX results, which showed that the modified samples contained the elements Ni (II), Cu (II), Mn (II), Co (II), Cr (III), and Fe (III), revealed the successful deposition of all treatments. The antibacterial and antifungal properties of the modified cotton complexes were investigated. The treated samples show antibacterial efficiency in higher results than antifungal properties and have great potential to be used as antibacterial textiles with high effectiveness. Moreover, the cotton fabric containing Ni (II) complex was exerted to be more reactive toward both bacteria and fungi than other metals modified cotton fabric complexes. According to the presented data, coated cotton fabrics are promising in antimicrobial textile applications.

## Figures and Tables

**Table 1:** FT-IR values of coated CF.

**Compound**	**ν (OH)**	**ν (CH_2_)**	**ν (C=O)**	**ν (C–O–C)**	**ν (C–O)**	**ν (M–O)**	**ν (M–N)**	**ν M–Cl**
AHZ-CF	3468–3191 br	2896, 2850 m	1652 m	1160, 1106 m	1053, 1028 s	-	-	-
Zn–AHZ-CF	3466–3184 br	2894, 2848 m	1648 m	1161, 1106 m	1053, 1028 s	591 w	518 m	434 m
Ni–AHZ-CF	3467–3188 br	2894, 2848 m	1651 m	1162, 1105 m	1053, 1028 s	590 w	518 m	434 w
Cu–AHZ-CF	3464–3191 br	2895, 2848 m	1651 m	1160, 1106 m	1053, 1028 s	592 w	518 m	435 w
Co–AHZ-CF	3468–3189 br	2895, 2848 m	1648 m	1160, 1105 m	1053, 1028 s	590 w	518 m	436 m
Cr–AHZ-CF	3464–3183 br	2895, 2846 m	1650 m	1161, 1105 m	1053, 1028 s	591 w	518 m	436 m
Mn–AHZ-CF	3461–3183 br	2896, 2851 m	1651 m	1160, 1106 m	1053, 1028 s	592 w	518 m	434 m
Fe–AHZ-CF	3461–3187 br	2896, 2851 m	1650 m	1160, 1106 m	1053, 1028 s	592 w	518 m	435 m

ν (wavenumber), S (strong), br (broad), m (medium), w (weak).

**Table 2:** The Antimicrobial activities of the blank cotton, AHZ-CF, and M-AHZ-CF.

Fabric Coating	Inhibition Zone (mm/cm Sample)		Inhibition Zone (mm/cm Sample)	
	Gram (+ve)	Gram (−ve)		
	*S. aureus*	*E. coli*	*C. albicans*	*A. flavus*
Blank	0	0	0	0
AHZ-CF	11	10	0	0
Zn−AHZ-CF	18	18	19	0
Ni−AHZ-CF	21	18	11	0
Cu−AHZ-CF	16	15	14	0
Co−AHZ-CF	14	14	0	0
Cr−AHZ-CF	12	13	0	0
Mn−AHZ-CF	13	12	0	0
Fe−AHZ-CF	11	0	0	0

**Table 3:** Results of add-on and tensile strength.

Cotton Sample	Add-On (%)	Tensile Strength (Kg f)
Blank	-	54.8 ± 0.2
AHZ-CF	2.17 ± 0.2	51.8 ± 0.2
Zn−AHZ-CF	4.5 ± 0.2	49.8 ± 0.2
Ni−AHZ-CF	5.97 ± 0.2	47.7 ± 0.3
Cu−AHZ-CF	5.75 ± 0.2	48.8 ± 0.2
Co−AHZ-CF	4.95 ± 0.2	48.50 ± 0.5
Cr−AHZ-CF	3.42 ± 0.2	50.67 ± 0.4
Mn−AHZ-CF	4.8 ± 0.2	49.32 ± 0.46
Fe−AHZ-CF	4.96 ± 0.2	48.3 ± 0.6

## Data Availability

All data that support the findings of this study are included within the article.
